# Modeling quorum sensing trade-offs between bacterial cell density and system extension from open boundaries

**DOI:** 10.1038/srep39142

**Published:** 2016-12-14

**Authors:** Mattia Marenda, Marina Zanardo, Antonio Trovato, Flavio Seno, Andrea Squartini

**Affiliations:** 1 SISSA, International School for Advanced studies, via Bonomea 265, 34136 Trieste, Italy; 2 Department of Agronomy, Animals, Food, Natural resources and Environment DAFNAE, Università di Padova, Viale dell’Università 16, 35020 Legnaro (Padova) Italy; 3 Department of Physics and Astronomy “Galileo Galilei” Università di Padova, Via Marzolo 8, 35131 Padova Italy; 4 INFN, National Institute for Nuclear Physics, Sezione di Padova, Via Marzolo 8, 35131 Padova, Italy; 5 CNISM, National Interuniversity Consortium for the Physical Sciences of Matter, Unità di Padova, Via Marzolo 8, 35131 Padova, Italy

## Abstract

Bacterial communities undergo collective behavioural switches upon producing and sensing diffusible signal molecules; a mechanism referred to as Quorum Sensing (QS). Exemplarily, biofilm organic matrices are built concertedly by bacteria in several environments. QS scope in bacterial ecology has been debated for over 20 years. Different perspectives counterpose the role of density reporter for populations to that of local environment diffusivity probe for individual cells. Here we devise a model system where tubes of different heights contain matrix-embedded producers and sensors. These tubes allow non-limiting signal diffusion from one open end, thereby showing that population spatial extension away from an open boundary can be a main critical factor in QS. Experimental data, successfully recapitulated by a comprehensive mathematical model, demonstrate how tube height can overtake the role of producer density in triggering sensor activation. The biotic degradation of the signal is found to play a major role and to be species-specific and entirely feedback-independent.

Quorum signaling and sensing have captured the interest of microbiologists for over two decades, being at the crossroads between cell physiology, population biology and mutual interactions in complex ecosystem communities. Most bacteria, colonizing a vast array of environments, build themselves a biofilm matrix, often through cooperative coordinated efforts; therefore it has become of particular applied interest to define cell-to-cell signal exchanges within such tridimensional organic matrices. The molecular basis for QS induction is the existence of a concentration threshold for diffusible signal molecules, above which they effectively bind to the corresponding receptors. This leads to an alteration of the gene expression pattern, and hence to a phenotype switch, regulated at the population level. The related cascade of events may include feedback mechanisms, that can either upregulate or downregulate signal production, and trigger signal biotic degradation^1^.

Different mathematical models to predict bacterial expression patterns were proposed. They take into account heterogeneity both at cellular and population level^2^,^3^,^4^,^5^, although a comprehensive understanding of QS ecological and evolutionary role is still lacking. QS original concept relates to an overall population census^6^, but other rationales have emerged, such as diffusion sensing by single cells^7^, clustering of bacterial aggregates within the biofilm matrix^8^,^9^, collective sensing and response to an heterogeneous environment^10^. Attempts to integrate such different views have been made, thereby connecting local with global population properties^11^,^12^. The QS response itself was recently shown to exhibit complex spatio-temporal patterns under flow^13^,^14^, whereas purely diffusing signals might sustain QS activation waves that travel across spatially extended colonies^15^,^16^.

Also in our own approach, the microbiological standpoint has been conjugated to the physical and mathematical data modeling. The importance of diffusion in shaping the signal concentration profile and in the consequent potential induction irrespective of cell density was addressed^17^. In this respect, the possible role of the relative cell positioning in biofilms as well as of population spatial extension was emphasized^17^. The presence of an absorbing boundary draining away the diffusing AHL molecules from the matrix was qualitatively shown to affect QS induction as importantly as cell density^18^.

Here, we systematically study the interplay between cell density and population extension away from an open boundary in the simplest possible system, with non-growing cell populations and without QS feedback affecting signal production. We devised a set up suited to compare ten different extensions in a ‘pan flute’ fashion, with open tubes of decreasing heights. They contain signal producer and reporter strains, embedded uniformly at different densities in a gel matrix. Tubes stand on a volume of the same gel acting as non-limiting space allowing free signal diffusion. In parallel a mathematical model was defined, consisting of a set of one dimensional lanes of different lengths where signal molecules are uniformly produced at a constant rate. These molecules are assumed to diffuse and to be biotically degraded. We consider one boundary as totally reflecting and the other one as adsorbing. Furthermore, the specific cascade of events leading from the QS response by the reporter strain to its experimental detection is taken into account in details in agreement with models previously described for different QS response systems^19^. The final goal was to derive the model parameters affecting the QS mechanism upon matching theoretical predictions with experimental data.

## Results

### Experimental dependence on spatial extension

**Figure 1:**
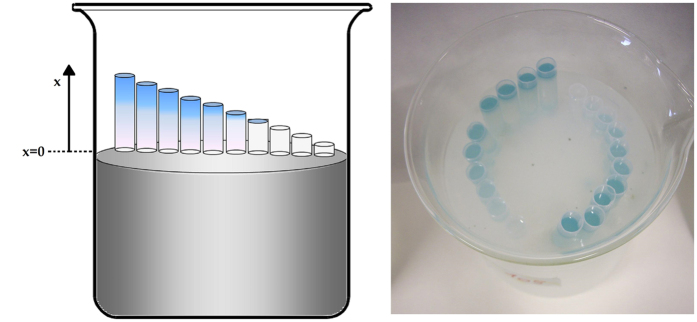
Pan Flute array for QS response extinction. Sketch and image of the technical set up to allow AHL molecules to flow out from the system under open conditions (i.e. via contact with a bulk of a 0,7% water based agarose gel) through the opened bottoms of the tubes containing the same gel with embedded cells of signal-producing *Rhizobium leguminosarum* A34, the reporter strain *Agrobacterium tumefaciens* NTL4 and the chromogenic substrate X-gal yielding the blue color upon the reach of AHL concentrations higher than quorum detection-effective threshold. The use of serial heights (from 2 to 20 mm), at which pipes are pre-cut, allows to test system extension effect. Embedding different producer cell concentrations enables to compare the cell density contribution to the observed activation phenotype. Notice that the shortest tubes of each row do not reach quorum-effective threshold (no blue color) in spite of containing the same cell density of the others in the same row. This reflects the fact that the balance between the produced molecules and those drained-off (diffusing into the bulk agarose gel from the open bottom) is shifted in favor of the latter with decreasing cylinder height.

Our model system consists in a set of agar cylinders of different heights, ranging from 2 to 20 mm, implanted in a beaker containing a large volume of agarose gel in water (see Methods and Fig. 1). In these cylinders *Rhizobium leguminosarum* bacterial cells producing AHL molecules are distributed with a uniform concentration *ρ*_*P*_. This set up is replicated for different values of the producer concentration *ρ*_*P*_. *Rhizobium* producer cells are mixed in all cases with a fixed uniform concentration *ρ*_*R*_ of the *Agrobacterium tumefaciens* reporter whose QS activation triggers the production of the *β*- gal enzyme. Preliminary tests with incubations of embedded cells in the same matrix and observations of rescued gel slices under brightfield microscopy had ascertained that the producer population does not grow under the present experimental conditions and cells maintain their spatial uniformity within the gel matrix over the experimental time.

**Figure 2:**
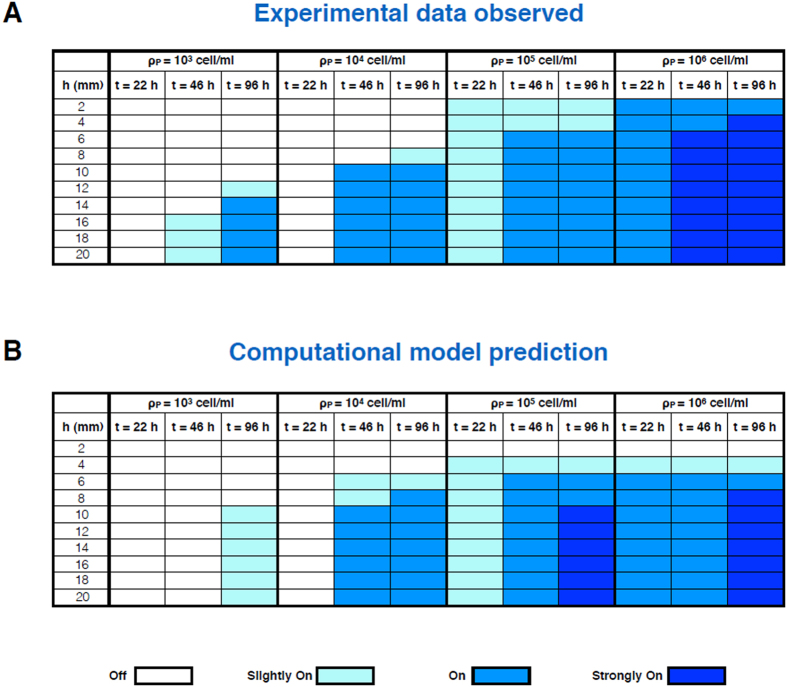
Mathematical model matching with experimentally observed data. Results of the reporter activation assay within systems with different extensions from the open boundary (cylinder heights) under free signal flow conditions (contact with the bulk water-based agarose gel) and different cell concentrations. **2 A**: actual observations recorded. **2B**: predictions given by the mathematical model described in methods. The four visual scores assigned in the experimental data are compared, in the mathematical model, to those derived from setting concentration thresholds conducive to effective induction of the *A tumefaciens* sensor (see Table 1 and Supplementary Table S1). While QS is by definition a dual on/off state when a single cell is considered, for phenotypes given by multiple cells in agar, the nuances (from slight to full) are dependent on the proportion of induced cells within the reporter population.

QS activation was monitored by the action of the produced enzyme on the X-gal substrate (see Methods) whose aglycone assumes a blue color upon enzymatic cleavage. For each set up, corresponding to a different producer concentration, QS activation is assessed for the 10 cylinder heights at three different times. Results are shown in Fig. 2A in the form of a color intensity table, where the color visual appearance is quantified by assigning four different (including white) nuances of blue.

### Biotic signal degradation is constitutive and species-specific

Experimental data show that equilibrium is reached at around 100 h, since the intensity of the observed colour at 120 h (data not shown) was unchanged with respect to the 96 h observation (Fig. 2). Upon assuming that the relaxation to equilibrium is governed by a simple rate-constant degradation kinetics of signal molecules, the observed effective rate appears considerably faster than that pertaining to the bare chemical degradation in the absence of bacteria, whose typical time was estimated to be of the order of 7 days^18^. At the same time, it is consistent with other findings where the effective degradation time was supposed to be around 10 hours^4^. In order to interpret these evidences, we tested whether the presence of bacteria might affect the effective degradation time due to biotic action upon the signal molecules. To evaluate this hypothesis we set up agar plates bearing embedded amounts of resuspended cells to different final concentrations. We tested non-AHL producing strains, and in particular we compared a *Rhizobium leguminosarum* field isolate (5ɣR2) and *E. coli* JM101. While *Rhizobium* is a taxon that utilizes quorum sensing in its physiology, *E. coli* is not. A drop of known amount of concentrated AHL was poured at the center of the plate and allowed to diffuse for 6 hours, after which an overlay of the *Agrobacterium* reporter was poured to monitor the concentration profile of signal molecules.

**Figure 3:**
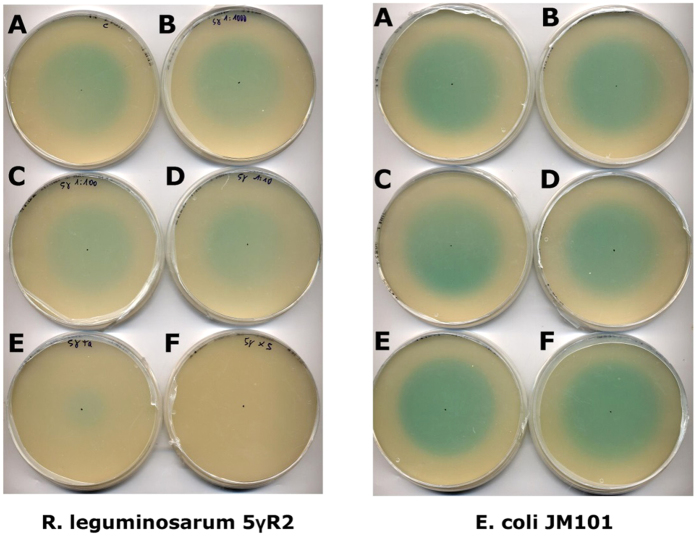
Species-specific and density dependent AHL degradation. Signal diffusion and degradation (drop of 100 ng in 10 μl of OHL at center diffusing 6 h) across different cell densities of non-producing bacteria. TY 0.7% agar Plates with embedded non-producers at increasing cell densities (**A**) control without bacteria: (**B**) 5.05 × 10^5 ^cells/ml, (**C**) 5.05 × 10^6 ^cells/ml, (**D**) 5.05 × 10^7 ^cells/ml, (**E**) 5.05 × 10^8 ^cells/ml, (**F**) 2.51 × 10^9 ^cells/ml). **Left panel**: *R. leguminosarum* 5ɣR2 (belonging to a species that uses AHL molecules for QS communication): **Right panel:** *E. coli* JM101 (belonging to a species that does not use AHL molecules nor QS communication). Reporter strain for both: overlay of *A. tumefaciens* NTL4 releasing the blue aglycone from X-gal upon QS induction.

As seen in Fig. 3, the presence of a QS-responding bacterial species, as *Rhizobium*, drastically reduces the concentration of AHL molecules for high cell densities confirming the presence of an effective biotic degradation, which dominates over the chemical one. This effect is not present for *E. coli*, which is known neither to produce nor to sense AHL molecules.

The efficient cell-density dependent mechanism of signal degradation in *Rhizobium* was furthermore proved to be independent of the newly-induced gene expression, as the same result was obtained when gentamycin, an antibiotic suppressing protein synthesis, was present in the agar medium hosting the embedded cells (Supplementary Fig. S1).

### Quantitative assessment of biotic signal degradation

**Figure 4:**
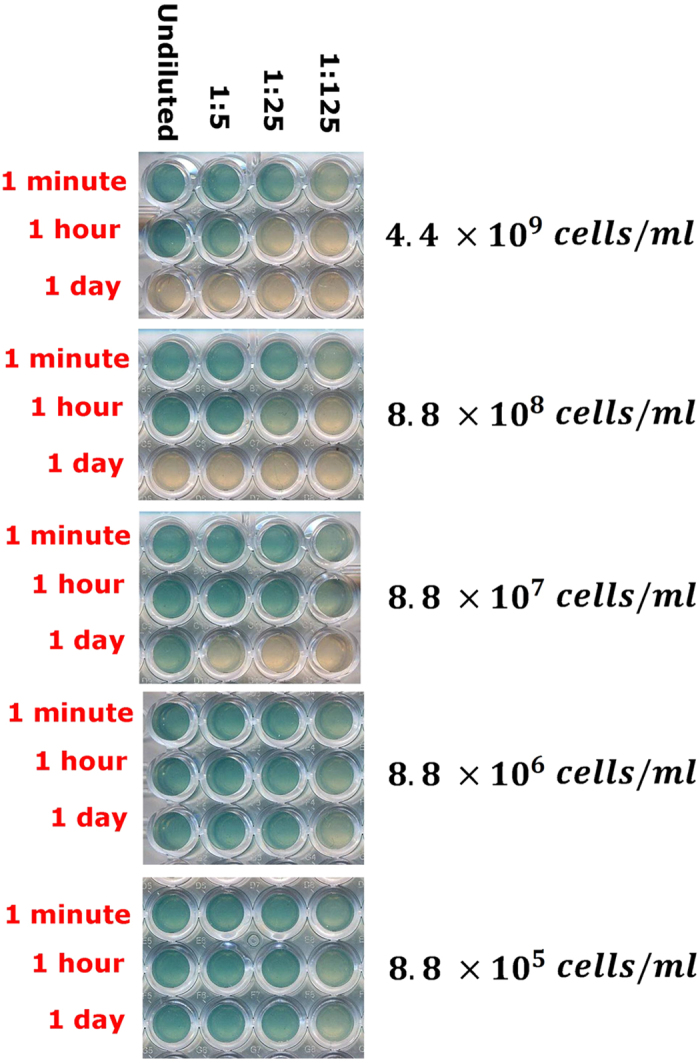
Temporal kinetics of signal degradation at different bacterial densities. After incubating for three temporal spans (1 min, 1 h, 1d) a fixed amount of OHL (550 ng) in eppendorf tubes containing the total numbers shown above of *R.leguminosarum* 5ɣR2 cells (non AHL-producer and effective degrader) centrifuged supernatants were tested with the sensor *A.tumefaciens* NTL4 in microtiter wells. An identical test with *E. coli* showed as expected no degradation (not shown). Negative controls (without bacteria) showed no degradation at all dilutions and times tested (not shown). By looking at the middle panel concentration (8.8 × 10^7 ^cells/ml), corresponding to the density range of *A.tumefaciens* sensor used in the “pan flute” experiment, the effective degradation time can be estimated by observing a similar response in the 1/25 dilution exposed for 1 hour and in the undiluted OHL exposed for 1 day. The response is analogously similar in the 1/125 dilution exposed for 1 hour and in the 1/5 OHL dilution exposed for 1/day. By assuming a single exponential degradation decay, we estimate an effective degradation time of 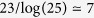 hours.

To achieve a finer measure of the biotic degradation mechanism effectiveness a further experiment was designed where a fixed amount of AHL molecules was mixed in physiological solution with different quantities of the non-AHL producing *Rhizobium* strain 5ɣR2. This suspension incubation was maintained for three different times: 1 minute, 1 hour and 1 day before being centrifuged to separate AHL molecules from bacterial cells. The supernatants, containing residual AHL, were diluted in serial fivefold steps, and fed to the *Agrobacterium* reporter in microtiter wells, to assess their still active concentrations. Negative controls were carried out incubating the AHL in plain physiological solution without the presence of bacteria. Results are presented in Fig. 4, showing that the effective degradation time is indeed directly related to the bacterial cell density. Moreover, the full quenching of the undiluted AHL concentration inducing capability is achieved in a one day exposure to at least 8.8 × 10^8^ cell/ml of a non-producing *Rhizobium* strain. On the other hand, the time resolution of Fig. 4 data does not allow to conclude that AHL degradation is a constant rate process.

This experiment allows also a quantitative comparison with the data obtained with the AHL producing *Rhizobium* strain A34 (Fig. 2A), if we consider the latter capable of degrading AHL as well. In this respect, at the concentration of 8.8 × 10^5^ cells/ml (used in the experiment shown in the lowest panel of Fig. 4), which in turn corresponds to the highest concentration used for the producer *Rhizobium* strain in the Pan Flute experiment (Fig. 2A), degradation is not detectable within one day. However, at higher concentrations (Fig. 4, higher panels), the effective degradation time becomes smaller and smaller. Now, it needs to be considered that also the *Agrobacterium* strain, used as embedded reporter in the Pan Flute experiment, is a Rhizobiaceae member and thence expected to be capable itself of signal degradation. Its constant concentration (10^8^ cells/ml) is of the same order of magnitude as the one of the *Rhizobium* strain used in Fig. 4 middle panel. A calculation based on the simplifying assumption of single exponential degradation kinetics allows to obtain in this case an effective biotic degradation time of roughly 10 hours (see caption of Fig. 4).

Taken together, the results shown in Figs 3 and 4, suggest that bacteria, which sense AHL molecules, are capable of degrading them at an effective rate strongly dependent on cell density. Moreover, we assume that the producer *Rhizobium* strain and the *Agrobacterium* reporter both degrade AHL with a similar quantitative dependence on cell density. In particular, in the height-dependent Pan Flute experiments the dominant effect is then actually due to the reporter strain whose density is the same in all cases and at least 100-fold higher than that of the producer strain. Therefore in our calculations (see Eq. (1) in Methods), biotic degradation by the producers is neglected 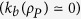 with respect to degradation by the reporters (*k*_*b*_(*ρ*_*R*_) = (10*h*)^−1^).

### Theoretical modeling of the dependence on system spatial extension away from an adsorbing boundary

Having reached a semi-quantitative understanding of the biotic degradation mechanism of AHL signal molecules, we modeled AHL dynamics in the height dependent experiments. We took into account diffusion in presence of relevant boundary conditions, constant and uniform production of AHL molecules, chemical and biotic AHL degradation due to the presence of bacterial cells (see Eq. (1) in Methods). Both degradation processes are assumed to be rate constant. We employed absorbing boundary conditions on the open face of the cylinders in order to speed up numerical simulations of Eq. (1). A first general prediction by the theoretical model is the existence of a characteristic distance from an absorbing boundary, given by 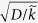, where *D* and 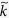 are the signal diffusivity and the effective degradation rate. QS response would strongly depend on the cell-colonized region extension only when the latter is less than the characteristic distance. Using parameter values reported in Table 1 one gets the characteristic distance of roughly 3 mm, in quantitative agreement with the experimental results shown in Fig. 2A.

The actual observation in the experiment (the appearance of the blue color) is related in a very indirect way to AHL concentration. As detailed in Methods, a fraction of reporters is activated depending on the ratio between the AHL concentration and the quorum threshold. Activated cells produce the *β*-gal enzyme, which then cleaves the X-gal substrate. Substrate diffusion is also considered for both the cleaved and un-cleaved populations. This entire process is described through Eqs (2), (3), and (4) in Methods. The whole set of equations can be numerically solved. The observable aspect that we directly compare with experimental data is the concentration of the cleaved substrate, reported in Table S1. This table can be translated into a four-states (including white) blue color intensity table upon introducing three different thresholds for cleaved substrate concentration. The thresholds were determined by optimizing the matching with the experimental results. The intensity table is plotted in Fig. 2B to be compared directly with the experimental results. Threshold concentrations for the cleaved substrate are reported in the caption of Table S1. The full list of parameters that we use, either by fixing or by optimizing them to better reproduce experimental data is reported in Table 1. Some parameters were taken from the literature: *D*_*s*_ (AHL diffusivity) and *k*_*e*_ (AHL chemical degradation rate) were derived in our previous work^18^; *D*_*s*_ was then assumed to be equal to *D*_*x*_ (substrate diffusivity, assumed to be the same for both the cleaved and the uncleaved substrate), due to the similar mass of the molecules (for spherical molecules, the ratio between the X-gal and the AHL diffusion constant would be 0.83, according to the Einstein-Stokes law); *k*_*B*_(*ρ*_*R*_) (AHL effective biotic degradation rate) is given by our actual experimental data (Fig. 4). The other parameters were derived in order to optimize the fit with experimental data. The value of *α* (AHL production rate) depends on the specific bacterial strain and while is not pre-set, is within the range indicated by different literature reports^4^,^17^; C^*^ (QS activation threshold for AHL concentration) is consistent with the value of our previous work^18^; the cooperativity parameter is set *m* = 1^20^; the enzymatic parameters are difficult to compare, since their values vary over different orders of magnitude depending on enzymes and substrates types^21^; moreover in our situation, dynamics is more complicated than standard Michaelis-Menten, since the enzyme is absent at the beginning, and is slowly produced once the quorum threshold is reached.

## Discussion

Comparing closed vs. adsorbing boundary conditions, we had previously shown that the effect of a nonreflecting boundary (pierced microtiter wells with a water flow underneath) was equivalent to that of a 100-fold lower producer cell density^18^. In the present report, in order to fully understand how the mechanics of the QS system depends on how far from an absorbing boundary the space colonized by bacteria extends, we adopt open conditions for the free outflow of diffusible signal molecules into a reservoir of non-limiting size (being much larger than the agar cylinders with embedded producer cells). The stepwise increasing height of the tube series allows to fine tune the analysis of the trade-offs between system extension away from the boundary and cell density, which turn out to be appreciable at a range of times and densities. For example (Fig. 2A) at 10^4^ cells/ml only a height of 10 mm or higher would allow the QS phenotype to be triggered; but a tenfold lower cell density would equally attain it for a height of at least 16 mm. The major breakthrough of the present paper is the definition of a mathematical model whose predictions very well approximate and fit actual observations (Fig. 2: compare 2A with 2B). Notably, all model ingredients are necessary in order to describe properly experimental data: 1) production, diffusion and biotic degradation of AHL molecules; 2) enzyme production via QS activation over AHL concentration threshold; 3) substrate diffusion and one-step enzymatic cleavage. In particular, AHL biotic degradation was taken into consideration with respect to several aspects. The diffusion of a pure AHL drop from the plate center against increasing cell densities in agar was analyzed (Fig. 3); data showed that: (a) taxa such as *Rhizobium*, in which QS is a widely used mechanism of communication, have a marked activity of signal interception and quenching of its capability to induce the sensor. Such attitude showed direct correlation with cell density in the medium and was present even in a strain that lacks the capability to produce its own AHL; (b) Species that are not known to respond nor adopt these QS mechanisms (*E. coli*) have no effect on blocking the molecule inducing potential for target species, even when used at extremely high density. Such outcome rules out the possibility that the quenching effect could be of non-specific nature as a generic partitioning into lipophilic structures as bacterial membranes or other cell components. This finding has also implications related to actual biofilms in the very frequent situation of mixed species, in which non-target microorganisms should therefore neither block nor attenuate signal output and exchanges between target ones. The subsequent experiment of signal extinction by Rhizobium (Fig. 4) clarifies the aspect of signal disappearance as a progressive event, calling for an enzymatic turnover, rather than for an instantaneous-irreversible form of uptake inside cells of proficient QS dialoguers. If that were the case, the extinction after 1 minute or 1 hour of exposure would have been supposedly as effective as the 1 day contact, while on the contrary one witnesses a regular time-related event of signal disappearance. In the theoretical modeling employed in this paper, a rate constant degradation kinetics, that does not change upon quorum activation, is employed for simplicity. A more refined modeling of signal enzymatic inactivation as a delayed QS-depending switch was proposed^22^.

On the other hand, another main result of the present paper is the recognition that the signal molecules biotic degradation may have constitutive nature. The aspect of AHL catabolism has assumed particular prominence in recent literature on the QS mechanism interpretation^23^ whereby questions have been put forward on the possible evolutionary benefits of such turnover and on its possible regulation or threshold dependent inducibility^24^. Hereby we provide evidence that the enzymatic degradation of the QS inducer is a basal, feedback-independent process. Its kinetics shows no differences between control cultures and those in which new protein synthesis and cell growth are irreversibly blocked by gentamycin (compare Fig. 3, left, and Supplementary Fig. S1). Moreover, feedback-independent enzymatic degradation is a key ingredient of the mathematical model that recapitulates the height-dependent behavior shown in Fig. 2.

The dependence of biotic signal degradation on bacterial cell density and the possibility of its feedback-dependent control (Quorum Quenching) in realistic QS systems^25^,^26^ intriguingly suggest that the trade-off studied in this paper may be amenable to a subtle regulation. A key role is indeed played by the interplay between signal diffusivity and degradation that sets the characteristic distance 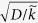 over which signal molecules can diffuse, on average, before they are degraded. The effect of an open boundary on QS response is then largely toned down for colonies extending further away from it (see the signal saturation for large cylinder heights in Fig. 2). Changing the effective biotic degradation rate would therefore be a way to regulate the boundary characteristic distance. This may be an important factor when devising anti-bacterial strategies based on signal inactivation^27^. Note that in the present system set up this further regulation level is not present, since the degradation rate is assumed to be the same in all experiments, being determined by the reporter strain density.

Our results on the trade-offs between system extension away from the boundary and cell density can be of particular relevance to infer the behavior and functioning of real microbial systems in nature as primarily the submerged biofilms. In those, typically developing on solid surfaces (rocks, plant roots, dental enamel, animal epithelia, tubing etc.), the underlying surface interface can be regarded as the signal-reflecting boundary, against which molecules as AHL would bounce back and concentration would build up (diffusion sensing). On the other hand, the side in contact with the flowing water would conversely offer a signal-adsorbing boundary which would drain it off and act against the reach of a quorum-effective concentration. Cell density and biofilm thickness (spanning in the hundreds of microns in natural ones) are therefore to be seen as tightly intertwined in setting the trigger for specific gene inductions ruled by the QS circuitry.

Different reports have dealt with the alternative views between spatial sensing and population census^1^ and considered factors as diffusion and cell clustering density as drivers of the process outcome. In our work we showed the dependency on a further aspect, which is the system spatial extension away from an open boundary, hereby represented by the height of a spatially homogeneous producing structure.

The model presented here highlights the crucial interplay of signal diffusion and signal degradation in tuning the influence of spatial extension on QS activation. The demonstration of the model suitability to predict the behavior of cultured QS bacteria are envisaged as useful cornerstones for the further unraveling of the bacterial communication paradigms, especially in submerged biofilms.

## Materials and Methods

### Mathematical model

In our model we mimic different bacterial densities in agar tubes as one-dimensional lanes of different heights *h* (see Fig. 1). One dimensionality is guaranteed by totally reflecting boundary conditions of the lateral sides of the tubes. The same boundary conditions are assumed for the base in contact with air^18^ at *x* = *h* whereas the boundary consisting in the base in contact with the agar beaker is assumed to be absorbing at *x* = 0. Our aim is to monitor the quantity of hydrolyzed X-Gal that we assume to be proportional to the intensity of the color experimentally observed. The full quorum sensing system is modeled as follows

#### Signal molecules dynamics

Since *R. leguminosarm* A34 does not display QS autoinduction^28^. The *Rhizobium* bacterial population at homogeneous concentration *ρ*_*P*_ produces AHL signal molecules with a constant rate production per cell *α*. The presence of the signal molecules is detected by the homogeneous overlayed suspension of the *Agrobacterium* sensor whose density is *ρ*_*R*_. The concentration 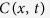
of AHL molecules at a distance *x* from the lower base at time *t* obeys to the following differential equation


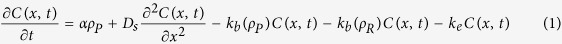


*D*_*s*_ is the diffusion constant of the signal molecules in agar and *k*_*e*_ is the chemical degradation rate in extracellular environment. 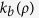
is the biological degradation rate due to the presence of the bacteria which, in general is an increasing function of bacterial cell density and is assumed to be the same for both producer and reporter strains. To satisfy boundary conditions we impose


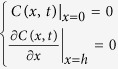


At stationarity the analytical solution of this equation is


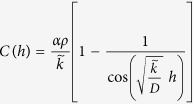


where 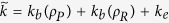.

In the limit of short lengths, e.g. 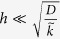. this implies 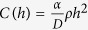.

#### Quorum sensing activation

The activation of the QS promoter leading to *β* − galactosidase production in the sensor strain occurs through a concerted binding of *m* signal molecules to the promoter itself. This can be modelled through a simple chemical reaction: 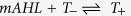. This means that at the equilibrium the fraction of activated bacterial cells is given by the Hill function:


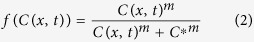


where C^*^ is the quorum sensing threshold. In particular we expect that the amount of enzyme Δ*E* produced in the small time interval Δ*t*, is equal to the promoter activity which we assume to be proportional to the Hill function through a parameter *A*, the maximum promoter activity^29^.

#### Production and dynamics of the tinted molecule

*β* − galactosidase enzyme (*E*) cleaves X-gal molecules (*S*) in order to produce the blue-colored molecules (*P*) which are observed. We model this enzymatic reaction as


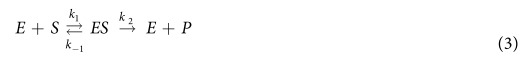


where the appropriate reaction rates are introduced. Both *S* and *P* obey the diffusion law with the same boundary conditions as the AHL molecules, but in principle with a different diffusion constant *D*_*x*_. The full process is therefore described by the following set of equations:


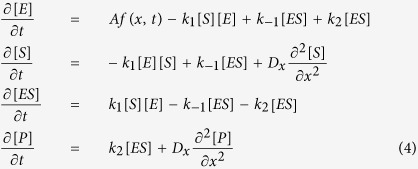


Equations (1,2,3,4) are numerically solved through finite difference methods^30^. As stated, *P(h, t*) is then compared to experimental results.

### System extension and cell density-dependent quorum activation assays

The rationale of this test was to have a signal-producing bacterial species along with a signal-reporting sensor strain, embedded together into a tridimensional system in a gel matrix suitable for signal diffusion. Different producer cell densities and different tube heights were tested. The bottom side of the tubes was in direct contact with a wide volume of the same gel (but without cells) in which the AHL molecules would dilute at rates which would compete with the production rates within the tube depending on the number of cells present and on the extension of the producing column (height of the tube). As AHL-producer we used *Rhizobium leguminosarum* bv. *viciae* A34^31^, which was grown in liquid TY medium^32^ at 30 °C. The strain does not possess beta-galactosidase activity. 2 ml from a 2-day old preculture were inoculated in 18 ml TY, after 24 h of growth, cells were centrifuged for 15′ at 5500 × g and resuspended in 10 ml of sterile saline solution (9.8 g/l NaCl). 100 μl were used for cell counting in a a Petroff-Hauser chamber in order to calculate aliquots to be mixed along with the reporter strain in the molten agarose suitable to deliver final concentrations of 10^3^, 10^4^, 10^5^, 10^6^ cells/ml upon calculations, the appropriate dilutions were made in sterile physiological solution in order to have the desired amount of cells in a volume of 200 μl.

The reporter of AHL quorum sensing molecules used was *Agrobacterium tumefaciens* NTL4 pZRL4^33^. The strain was grown in liquid AB medium^34^ supplemented with 30 μg/ml gentamycin at 30 °C. 5 ml of an overnight preculture, were inoculated in 45 ml of AB and grown for 18 h under the same conditions. For each of the *Rhizobium* producer cell concentration suspensions, 6.9 ml of this *A.tumefaciens* culture were subsequently mixed with 13.1 ml of molten AB agarose (10 g/l) previously cooled to 42 °C, 200 μl of the above mentioned Rhizobium cells resuspensions in physiological solution, 60 μl of a 20 mg/ml X-gal (5-bromo-4-chloro-3-indolyl-beta-D-galactopyranoside) stock solution, for a final concentration 60 μg/ml. The resulting suspension was rapidly pipetted into a series of sterile polypropylene open-ended plastic tubes sections, pre-cut to accommodate gel volumes to top heights from 2 mm to 20 mm, and fitted standing over a beaker containing 500 ml of agarose (Sigma-Aldrich, St Louis MO, USA) 0,7% solution in distilled water) gel. The beaker top was covered with parafilm and the system was incubated at 20 °C periodically inspecting the appearance of the blue color.

### Signal diffusion tests across different cell densities of non-producing bacteria

The test was devised to verify the extent of possible quenching/degradation exerted by non-producing bacterial cells encountering a diffusing gradient of a pure AHL molecule. Different cell densities and bacterial species were tested. The chosen AHL was OHL (N-Octanoyl-L-Homoserine lactone, Fluka Chemie Gmbh Buchs, Switzerland, molecular weight 227.3

As non-producers of AHL, a strain of *Rhizobium leguminosarum* bv. *viciae* 5ɣR2 isolated from pea nodules (Squartini, unpublished) and *Escherichia coli* JM105^35^ were used. Both strains, are also negative in beta-galactosidase activity, which avoids interference in the X-gal based assay. Cells were grown in TY at 30 °C (*R. leguminosarum*) or 37 °C (*E. coli*) and from the precultures 38 ml were inoculated in 342 ml TY, after 24 h of growth, 300 ml of culture were centrifuged for 15′ at 5500 × g and resuspended in 20 ml of TY. 100 μl were used for cell counting in a Petroff-Hauser chamber. Dilutions in TY were made in order to be able to mix 33.75 ml of molten TY agar (0.7% final concentration) cooled to 42 °C with 16.25 ml of cell suspension, to provide the following final cell concentrations: 5.05 × 10^5^, 5.05 × 10^6^, 5.05 10^7^, 5.05 × 10^8^, 2.51 × 10^9^ (cells/ml). Each plate contained 20 ml of agar medium with cells suspension and 60 μg/ml X-gal. Control plates were made containing only TY agar/X-gal without embedded bacteria.

Upon agar solidification 100 ng of OHL were dispensed at the center of the plates (10 μl of a 10 ng/μl solution). The OHL was let to diffuse through the agar embedded with cells and after 6 hours, an overlay of *Agrobacterium tumefaciens* NTL4 reporter strain suspension was poured over the previously laid agar surface. The reporter was grown in liquid AB medium supplemented with 30 μg/ml gentamycin at 30 °C. 10 ml of an overnight preculture, were inoculated in 100 ml of AB and grown for 18 h under the same conditions. 67 ml of this culture were subsequently mixed with 133 ml of molten AB agarose (10 g/l) containing 30 μg/ml gentamycin and 60 μg/ml X-gal (5-bromo-4-chloro-3-indolyl-beta-D-galactopyranoside) which was previously cooled to 42 °C. 15 ml of overlay were poured in the petri dishes. Images were acquired with an Epson Perfection 1240 U digital flatbed scanner after 21 hours of exposure to the reporter overlay.

### Temporal kinetics of signal degradation at different bacterial densities

In order to verify the extent of linearity in the time-dependent degradation of the AHL molecules by degradation-proficient bacteria, and calculate its kinetics, an experiment was set up to expose a given amount of OHL to cells suspended in physiological solution, incubating them for three increasing time lapses. 550 ng of OHL in 50 μl of sterile distilled water were added to 1620 μl of cell suspensions from freshly grown cultures resuspended in physiological solution, at the concentrations of 7.35 × 10^9^, 1.47 × 10^9^, 1.47 × 10^8^, 1.47 × 10^7^, 1.47 × 10^6^ total cells, in sterile 1.5 ml Eppendorf tubes. Suspensions were incubated at 22 °C for 1 min, or 1 h or 24 h, centrifuged in an Eppendorf benchtop centrifuge for 5′ at 13,000 × g. Supernatants (1 ml) were withdrawn and frozen. A 96-well microtiter plate containing 200 μl of *A.tumefaciens* suspension in AB medium with X-gal, prepared as described in the previous paragraph was set up. Thawed supernatants and three of their serial fivefold dilutions were dispensed (10 μl per well). After 24 h the digital image was acquired by a scanner as described above.

## Additional Information

**How to cite this article**: Marenda, M. *et al*. Modeling quorum sensing trade-offs between bacterial cell density and system extension from open boundaries. *Sci. Rep.* **6**, 39142; doi: 10.1038/srep39142 (2016).

**Publisher's note:** Springer Nature remains neutral with regard to jurisdictional claims in published maps and institutional affiliations.

## Supplementary Material

Supplementary Information

## Figures and Tables

**Table 1:** List of the parameters used for data fitting.

Signal molecules dynamics		
AHL production rate per cell	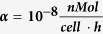	**fit**
AHL diffusion constant	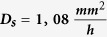	**[18]**
AHL chemical degradation rate in extracellular environment	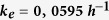	**[18]**
AHL biological degradation rate due to *Rhizobium*	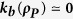	**exp**
AHL biological degradation rate due to *Agrobacterium*	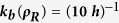	**exp**
*Rhizobium* cellular density	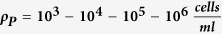	**exp**
*Agrobacterium* cellular density	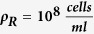	**exp**
**Quorum Sensing activation**		
Cooperativity parameter	*m* = 1	**[20]**
Quorum Sensing threshold	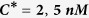	**fit**
**Production and dynamics of the tinted molecule**		
Maximum promoter activity	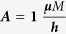	**fit**
Reaction rate *E* + *S* → *ES*	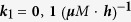	**fit**
Reaction rate *ES* → *E* + *S*	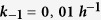	**fit**
Reaction rate *ES* → *E* + *P*	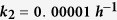	**fit**
X-Gal diffusion constant	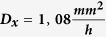	**ass.**
X-Gal initial concentration	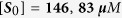	**exp**

The reason for parameter choice is specified: optimization of the matching between theoretical and experimental data (fit), experimental set up conditions or other experimental results provided here (exp), literature reference ([…]), physical assumption (ass.).
