# Supplementary material for: Modeling quorum sensing trade-offs between bacterial cell density and system extension from open boundaries
Source: Sci Rep. 2016 Dec 14;6:39142. doi: 10.1038/srep39142 (PMC5155435; doi:10.1038/srep39142)
Supplement: Supplementary Information [file srep39142-s1.pdf]

Modeling quorum sensing trade offs between bacterial cell density and system extension from open boundaries.

Mattia Marenda[1], Marina Zanardo[2], Antonio Trovato[3], Flavio Seno[3], and Andrea Squartini[2]*

[1] SISSA, International School for Advanced studies, via Bonomea 265, 34136 Trieste, Italy; [2]Department of Agronomy, Animals, Food, Natural resources and Environment DAFNAE, Università di Padova, Viale dell'Università 16, 35020 Legnaro (Padova) Italy;
[3] CNISM and Department of Physics and Astronomy "Galileo Galilei" *Università di Padova,* Via Marzolo 8, 35131 Padova Italy;

Correspondence to: Andrea Squartini, Department of Agronomy Animals, Food, Natural Resources and Environment, DAFNAE, University of Padova, Legnaro (Padova) Italy. squart@unipd.it

	$\rho_P = 10^3\ cell/ml$			$\rho_P = 10^4\ cell/ml$			$\rho_P = 10^5\ cell/ml$			$\rho_P = 10^6\ cell/ml$		
$Height\ (mm)$	$t = 22\ h$	$t = 46\ h$	$t = 96\ h$	$t = 22\ h$	$t = 46\ h$	$t = 96\ h$	$t = 22\ h$	$t = 46\ h$	$t = 96\ h$	$t = 22$	$t = 46\ h$	$t = 96\ h$
2	0,0004	0,0003	0,0002	0,0034	0,0028	0,0020	0,0234	0,0212	0,0190	0,0612	0,0581	0,0551
4	0,0124	0,0231	0,0177	0,1103	0,1867	0,1715	**0,5285**	**0,7249**	**0,7108**	**0,8960**	**1,1198**	**1,1063**
6	0,0262	0,0964	0,1883	0,2261	**0,8021**	**1,2740**	**0,9846**	**2,9439**	**3,6466**	**1,5871**	**4,1119**	**4,8270**
8	0,0344	0,1557	0,4906	0,2913	**1,2765**	**3,6404**	**1,2108**	**4,6967**	**9,5695**	**1,9083**	**6,6827**	**11,9550**
10	0,0385	0,1966	**0,7298**	0,3243	**1,5920**	**5,7469**	**1,3193**	**5,6887**	**16,6853**	**2,0602**	**8,0258**	**20,6354**
12	0,0405	0,2227	**0,9158**	0,3398	**1,7911**	**7,1922**	**1,3689**	**6,2895**	**22,5610**	**2,1286**	**8,8179**	**28,3226**
14	0,0414	0,2388	**1,0551**	0,3467	**1,9120**	**8,2474**	**1,3903**	**6,6470**	**26,3254**	**2,1583**	**9,2863**	**33,9018**
16	0,0418	0,2483	**1,1562**	0,3495	**1,9832**	**9,0074**	**1,3991**	**6,8546**	**28,6682**	**2,1703**	**9,5575**	**37,4401**
18	0,0419	0,2537	**1,2281**	0,3506	**2,0238**	**9,5456**	**1,4023**	**6,9719**	**30,2567**	**2,1749**	**9,7107**	**39,6219**
20	0,0420	0,2567	**1,2784**	0,3510	**2,0463**	**9,9212**	**1,4037**	**7,0365**	**31,3492**	**2,1765**	**9,7950**	**41,0583**

Off: $C < 0,5\ nM$ ***Slightly on:*** $0,5\ nM < C < 1,5\ nM$ ***On:*** $1,5\ nM < C < 10\ nM$ ***Strongly on:*** $C > 10\ nM$

Table S1: Results of the mathematical model describing Pan Flute experiments. Cleaved X-Gal product molecules concentration (nM). Values are calculated for different *Rhizobium* producer strain densities ρ_P, in different times t and for different system heights h. In caption, colour thresholds values (linked to cleaved X-Gal concentrations).

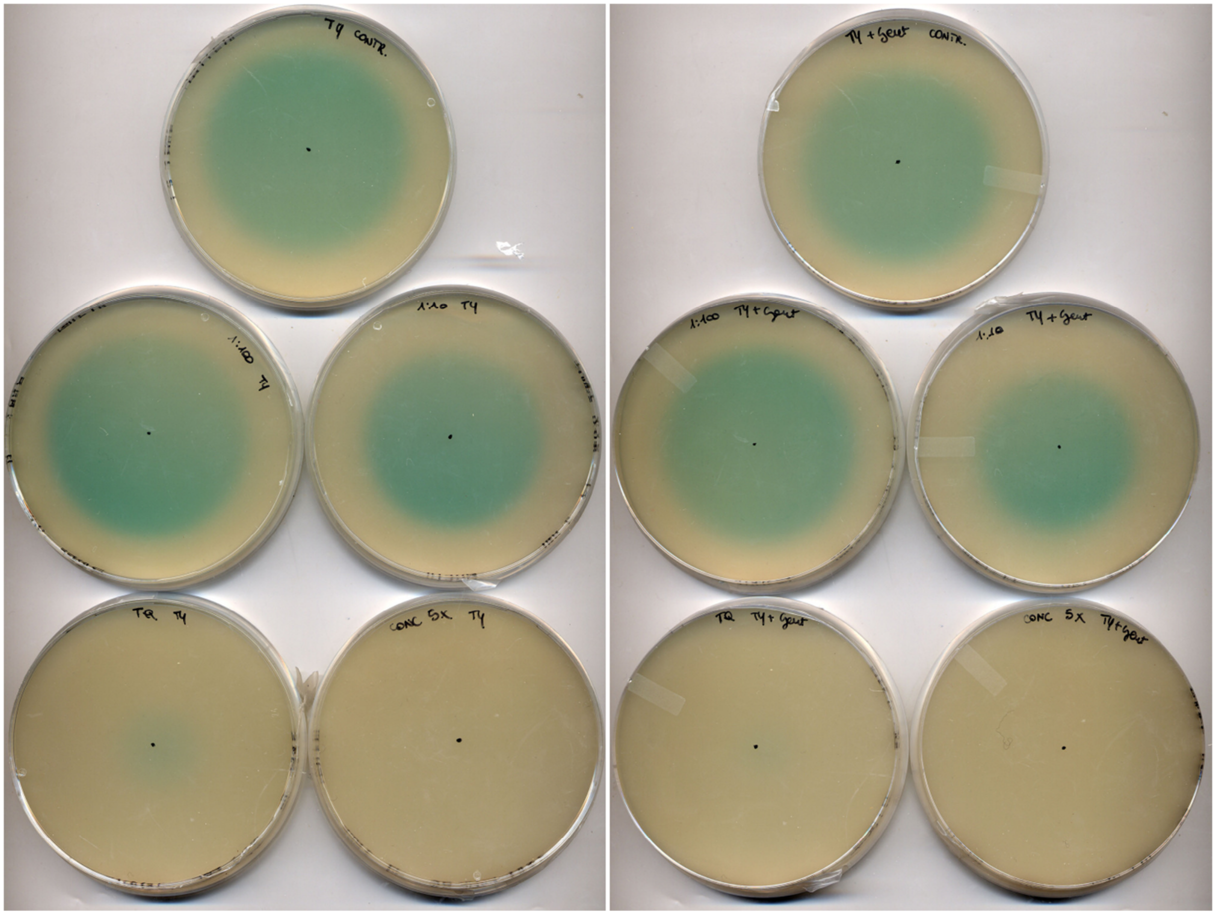

Fig.S1. Signal diffusion and degradation (drop of 100 ng in 10 µl of OHL at center diffusing 6 h) across different cell densities of the non-producing bacterium *R.leguminosarum* 5ɣR2. TY 0.7% agar Plates with embedded non-producers at increasing cell densities. **Left panel**: plain medium; **Right panel**: supplemented with 30 µg/ml gentamycin. Cell densities, From the top, descending : control without bacteria; 5,05 x 10^6 cells/ml, 5,05 10^7 cells/ml, 5,05 x 10^8 cells/ml, 2,51 x 10^9 cells/ml). Reporter strain for both: overlay of *A. tumefaciens* NTL4 releasing blue X-gal upon QS induction.

The *Rhizobium* strain was previously ascertained to be Gm-sensitive and under these conditions its cells are uncapable of cell division as well as blocked in protein synthesis. The equal behavior of the blue-colored phenotype demonstrates the constitutive nature of AHL biotic degradation.
